# Quantitative cardiovascular magnetic resonance findings and clinical risk factors predict cardiovascular outcomes in breast cancer patients

**DOI:** 10.1371/journal.pone.0286364

**Published:** 2023-05-30

**Authors:** Jennifer M. Kwan, Amit Arbune, Mariana L. Henry, Rose Hu, Wei Wei, Vinh Nguyen, Seohyuk Lee, Juan Lopez-Mattei, Avirup Guha, Steffen Huber, Anna S. Bader, Judith Meadows, Albert Sinusas, Hamid Mojibian, Dana Peters, Maryam Lustberg, Sarah Hull, Lauren A. Baldassarre

**Affiliations:** 1 Section of Cardiovascular Medicine, Yale School of Medicine, New Haven, CT, United States of America; 2 Department of Biostatistics, New Haven CT Yale School of Public Health, New Haven, CT, United States of America; 3 Allegheny General Hospital, Pittsburg, PA, United States of America; 4 Lee Memorial Hospital, Fort Meyers, FL, United States of America; 5 Medical College of Georgia, Augusta, Georgia, United States of America; 6 Department of Radiology, Yale School of Medicine, Section of Medical Oncology Yale School of Medicine, New Haven, CT, United States of America; 7 Cardiology, Yale School of Medicine, New Haven, CT, United States of America; University of Bologna, ITALY

## Abstract

**Background:**

Cardiac magnetic resonance (CMR) global longitudinal strain and circumferential strain abnormalities have been associated with left ventricular ejection fraction (LVEF) reduction and cardiotoxicity from oncologic therapy. However, few studies have evaluated the associations of strain and cardiovascular outcomes.

**Objectives:**

To assess CMR circumferential and global longitudinal strain (GLS) correlations with cardiovascular outcomes including myocardial infarction, systolic dysfunction, diastolic dysfunction, arrhythmias and valvular disease in breast cancer patients treated with and without anthracyclines and/or trastuzumab therapy.

**Methods:**

Breast cancer patients with a CMR from 2013–2017 at Yale New Haven Hospital were included. Patient co-morbidities, medications, and cardiovascular outcomes were obtained from chart review. Biostatistical analyses, including Pearson correlations, competing risk regression model, and competing risk survival curves comparing the two groups were analyzed.

**Results:**

116 breast cancer with CMRs were included in our analysis to assess differences between Anthracycline/Trastuzumab (AT) (62) treated versus non anthracycline/trastuzumab (NAT) (54) treated patients in terms of imaging characteristics and outcomes. More AT patients 17 (27.4%) developed systolic heart failure compared to the NAT group 6 (10.9%), p = 0.025. Statin use was associated with a significant reduction in future arrhythmias (HR 0.416; 95% CI 0.229–0.755, p = 0.004). In a sub-group of 13 patients that underwent stress CMR, we did not find evidence of microvascular dysfunction by sub-endocardial/sub-epicardial myocardial perfusion index ratio after adjusting for ischemic heart disease.

**Conclusions:**

In our study, CMR detected signs of subclinical cardiotoxicity such as strain abnormalities despite normal LV function and abnormal circumferential strain was associated with adverse cardiovascular outcomes such as valvular disease and systolic heart failure. Thus, CMR is an important tool during and after cancer treatment to identity and prognosticate cancer treatment-related cardiotoxicity.

## Introduction

In the United States, breast cancer is the most common cancer diagnosed among women [1] and, after lung cancer, it is the second leading cause of cancer death among women [1]. However, due to cancer therapies such as radiation, chemotherapy, human epidermal growth factor receptor-2 (HER-2) inhibitors and more recently, immunotherapy, more patients have been surviving breast cancer [2, 3].However, cardiovascular disease remains a leading cause of death in breast cancer patients [4, 5]. This could be in part due to underlying cardiovascular risk factors that are concurrent with cancer risk factors, such as smoking and obesity, but could also be due to cancer therapies themselves [6–8].

Cancer treatment-related cardiac dysfunction (CTRCD) has been well documented in the literature [9]. In a recent study, breast cancer patients treated with trastuzumab had a three-fold higher risk of left ventricular dysfunction as compared to those who did not receive trastuzumab [10]. Similarly, anthracyclines have also been documented to cause irreversible cumulative dose dependent cardiotoxicity in the form of left ventricular dysfunction in breast cancer patients [11, 12].

Monitoring for adverse cardiovascular outcomes associated with oncologic therapies is crucial in improving long term outcomes of breast cancer patients. Cardiac imaging plays a critical role during and after cancer treatment in identifying CTRCD. Recent European Society of Cardiology (ESC) guidelines recommend baseline echo using 3D analysis of left ventricular ejection fraction (LVEF) for those undergoing anthracycline therapy, regardless of cardiovascular risk, and use of CMR imaging as second line if echo is of suboptimal quality [13–15]. Ischemia, microvascular dysfunction and chemotherapy induced cardiotoxicity are potential diagnoses when a breast cancer patient presents with cardiac symptoms or cardiomyopathy after receiving chemotherapy [16]. Although there is equivocal evidence to support the use of stress testing in cancer patients prior to chemotherapy [17, 18], monitoring for cardiovascular toxicities can help decide whether a patient needs cardioprotective medications and/or adjustment or cessation of cancer treatment [13]. Breast cancer patients, particularly those on HER2 inhibitors, are recommended to receive cardiac monitoring at baseline, subsequently at 3 month intervals, and post therapy [19, 20], yet adherence is highly variable [10]. While studies like these have documented the incidence and prevalence of CTRCD in breast cancer patients, as well as cardiac monitoring adherence, these studies may lack comprehensive information on patient comorbidities, patient demographics, and other clinical characteristics, which may help explain the risk of cardiovascular disease in these patients. However, it is well established that age, hypertension, baseline low ejection fraction, and prior anthracycline use are risk factors for cardiotoxicity [21]. Studies have shown the importance of cardiac monitoring in this population and have provided some reference imaging parameters for detection of CTRCD [22–26] such as strain imaging in addition to left ventricular ejection fraction [27–29]. Recent randomized controlled trials suggest incorporation of strain image guided monitoring approach for initiation of cardioprotective therapies may help reduce reduction in LVEF drop compared to LVEF image guided approach, and this is reflected in the 2022 ESC guidelines, which recommend global longitudinal strain (GLS) for baseline and serial monitoring for those undergoing cardiotoxic chemotherapy [15, 30]. For cancer patients, CMR has shown value in the context of cardiomyopathy evaluation by impacting clinical diagnosis and management [31]. CMR has also been used to evaluate for LVEF and strain, using feature tracking strain and strain encoded (SENC) techniques , amongst others, for detection of subclinical cardiotoxicity [32]. CMR also holds value for tissue characterization, including assessment of T2-weighted images and T1-weighted imaging, including delayed gadolinium enhancement, to evaluate for edema and fibrosis [33–35]. Abnormal strain by echocardiography was associated with increased risk of major adverse cardiovascular outcomes in patients on immunotherapy [36]. More recently, echo strain and CMR strain have been compared in a prospective cohort of 47 patients undergoing cardiotoxic chemotherapy and, although there was a weak correlation between the two modalities, strain was predictive of future decline in LVEF [37]. However, in a retrospective study of 50 patients who had both CMR and echo done within a mean of 8.5 ± 9.8 days, there was a good correlation in strain between echo (speckle tracking) and CMR (both feature tracking and SENC) r = 0.7 [38]. Both longitudinal and circumferential strain by CMR are recommended by the 2022 ESC guidelines for evaluation of cardiotoxicity [15]. CMR is a useful tool to provide additional information about tissue characterization using late gadolinium imaging and parametric mapping techniques. There have been various studies demonstrating the use of parametric quantification techniques like extracellular volume (ECV) to determine anthracycline-induced cardiotoxicity [39], and there are ongoing trials planning to evaluate these parameters of CMR in anthracycline cardiotoxicity [40]. However, less is known about the relationship of CMR strain, function, and volumetrics with longer term cardiovascular outcomes.

The objective of our study is to assess CMR parameters and cardiovascular risk factors for prediction of cardiovascular outcomes in breast cancer patients, comparing those treated with anthracyclines and/or trastuzumab (AT) vs those who were not treated with anthracyclines nor trastuzumab (NAT).

## Methods

### Patient sample and data extraction

**Fig 1:**
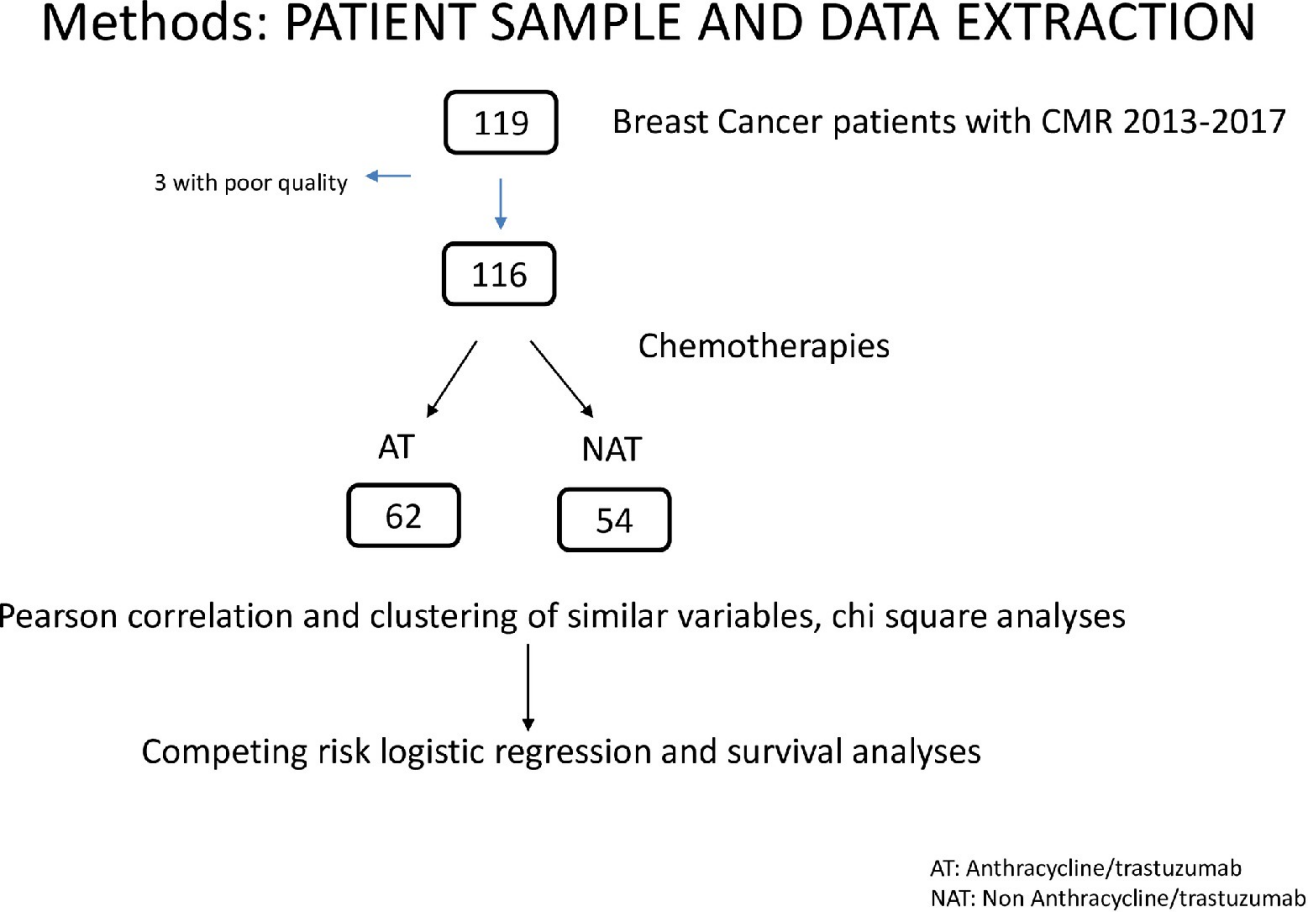
Data extraction and patient algorithm.

Patients in the sample included all breast cancer patients who underwent a CMR at Yale New Haven Hospital from 2013 to 2017, and chart review was conducted as far back as 1990 to assess for patient co-morbidities. The sample consisted of 116 patients with a history of breast cancer. Of these 116 patients, 62 were in the AT group, while 54 were in the NAT group. Patients were excluded if they had poor quality/nondiagnostic CMRs (n = 3). **Fig 1** depicts how the sample of patients was selected. A small subset (n = 13) had stress CMR performed during chemotherapy treatment. Information on patient comorbidities, demographics, cancer treatments, cardiac medications, and cardiovascular outcomes were obtained by chart review from the electronic health record system at Yale New Haven Hospital. The institutional review board of Yale University School of Medicine reviewed and approved this research.

### CMR protocol and post processing

CMR imaging was performed on 1.5 and 3T scanners (Siemens, Erlangen, Germany) with standard image acquisitions. Steady state free precession (SSFP) cine imaging [repetition time (TR) = 3 ms, echo time (TE) = 1.5 ms, flip angle (FA) = 60°, 30 cardiac phases, 1.4x 1.4 x 8 mm3 resolution) with retrospective ECG gating was acquired in the two-chamber, three-chamber, and four-chamber views, and in contiguous short axis slices of the left ventricle.

Short axis T2W black-blood fast spin echo images were used [field of view (FOV) = 360mm×270mm, TE = 65 ms, slice thickness = 8 mm, FA = 90°, bandwidth = 781 Hz/pixel, 0.8 mm resolution]. T2W mapping used T2W-prepared balanced steady-state free precession (bSSFP) single shot images acquired in 9 heart beats (TEs = 0 ms, 25 ms, 45 ms) with FOV = 360 mm x 270 mm, slice thickness = 8 mm, FA = 30°, parallel imaging = generalized autocalibrating partially parallel acquisition (GRAPPA 2), bandwidth = 1395Hz/pixel). The T2W maps were generated using an exponential fit (image resolution 1.5mm×1.5mm x 8mm). Late gadolinium enhancement (LGE) evaluation was performed 8–10 min after administration of 0.2 mmol/kg gadolinium contrast agent. After performing an inversion scout sequence, LGE imaging was obtained using inversion recovery gradient echo (TR/TE/θ = 6 ms/3.16ms/25°, 1.64 x 1.64 x 8 mm).

**Fig 2:**
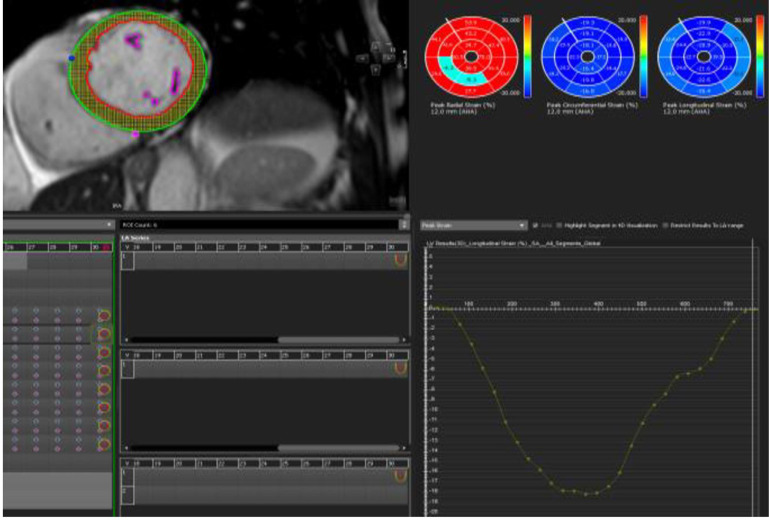
Example of feature tracking using CVI42, endocardium (red) and epicardium (green) contours manually traced on the SSFP cine short axis stack, long axis in cine 4 chamber, 2 chamber and LVOT series during end diastole and systole. The yellow dots between the red and green contours correspond to the myocardium which is tracked during systole for feature tracking GLS.

Quantitative analysis for volumes, function, and feature tracking strain for 3D LV global longitudinal strain (GLS), global circumferential strain (GCS), and global radial strain (GRS) was performed using SSFP cine in short axis, 2-chamber, 3-chamber, and 4-chamber views with CMR42 (CVI42, v5.6 –v 5.10, Calgary, Canada). This tissue tracking module recognizes patterns of features in the image that can be tracked in successive images (**Fig 2).** Normal MRI reference ranges are featured in **S1 Table in S1 File** [41].

**Fig 3:**
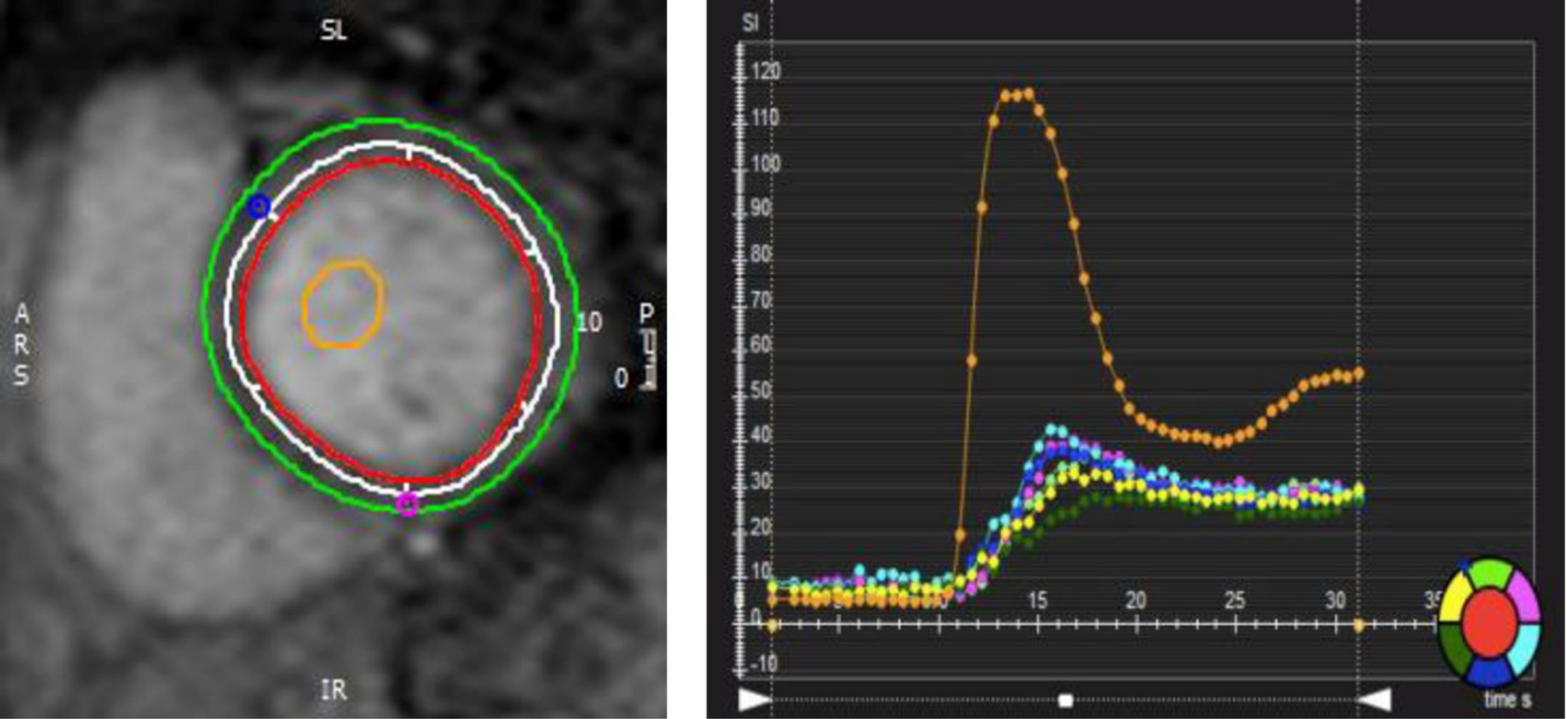
**a.** First-pass vasodilatory stress with regadenoson. b-endocardial layer to be analyzed as shown. Endocardium (red) and epicardium (green) contours were manually outlined for sub-endocardial/sub-epicardial myocardial perfusion index ratio analysis. To normalize to the arterial input function, a region of interest was drawn inside the LV blood pool (orange). The white contour circumscribes the subendocardial layer to be analyzed as shown. **b.** Myocardial signal intensity over time curve of the six myocardial segments and the LV blood pool, represented by the color palette to the bottom right. Myocardial perfusion index is the ratio between the maximum slope of one segment and the maximum slope of the blood pool (orange curve). All six segments were averaged to obtain a global sub-endocardial/sub-epicardial myocardial perfusion index ratio.

Stress CMR protocol included first-pass vasodilatory stress-only perfusion (ssGRE) with regadenoson 0.4 mg and injection of 0.05 mmol/kg contrast agent, injected 90 seconds and 8 minutes after stress agent. The perfusion scan protocol was: field of view (FOV) = 360mm×270mm, TR/TE = 6/2 ms, slice thickness = 8 mm, FA = 20°, bandwidth = 781 Hz/pixel, spatial resolution 3x3mm, 3 slices per heart-beat. Quantitative parameters were processed (CVI42, v5.6.4, Calgary, Canada) for LV volume and function, feature tracking GLS (**Fig 3).** Endocardium and epicardium contours were manually outlined for sub-endocardial/sub-epicardial myocardial perfusion index ratio analysis. To normalize to the arterial input function, a region of interest was drawn inside the LV blood pool **(Fig 3A).** Myocardial signal intensity over the time curve of the six myocardial segments and the LV blood pool are represented by the color palette to the bottom right. Myocardial perfusion index is the ratio between the maximum slope of one segment and the maximum slope of the blood pool (orange curve). All six segments were averaged to obtain a global sub-endocardial/sub-epicardial myocardial perfusion index ratio **(Fig 3B).**

### Statistical methods

Chi-square or Wilcoxon test was used to compare demographic and clinical features between AT and NAT groups. Mean and standard deviation were calculated for echo and CMR variables. A two-sample *t*-test was used to compare means for the imaging parameters, which were normally distributed.

Pearson correlations were used to examine relationships between demographic and clinical variables and CMR imaging parameters. All statistical analyses were performed using SAS 9.4, Graphpad or R.

Data was obtained from electronic medical records via chart review to determine diagnoses and pre-existing co-morbidities. The index date was the time of initiating chemotherapy, and outcomes were assessed at 3 years or more from initiation of chemotherapy. As the data includes patient follow up for a substantial period of time, a competing risk regression model was used to evaluate the risk of subsequent systolic heart failure (HF), diastolic HF, coronary artery disease, myocardial infarction, peripheral arterial disease, thromboembolism, arrhythmia , and valvular disease (greater than mild mitral regurgitation, aortic regurgitation, aortic stenosis, mitral stenosis, tricuspid regurgitation, ie moderate or severe) starting from time of oncologic therapy initiation. Systolic dysfunction was defined as LVEF <50%, and diastolic dysfunction included grades 1–3 of diastolic dysfunction by echo. Myocardial infarction (MI) was defined by having non-ST elevation MI or ST elevation MI. Peripheral arterial disease is defined as having clinically significant peripheral artery disease where the patient had symptoms. Arrhythmia included bradycardia that was symptomatic or required intervention, heart block, atrial fibrillation, atrial flutter, supraventricular tachycardia, and ventricular arrhythmias). Valvular disease included greater than mild mitral regurgitation, aortic regurgitation, aortic stenosis, mitral stenosis, or tricuspid regurgitation. Thromboembolism is defined as having imaging confirmed deep venous thrombosis or pulmonary embolism. All diagnoses were clinically determined and documented in clinical notes. Results are expressed in hazard ratios (HRs) for each predictor, its 95% confidence interval (CI), and corresponding p-value.

The cardiotoxicity analysis first involved a univariate analysis on nineteen different predictors and across six different outcomes. The predictors include age, diabetes (DM), peripheral artery disease (PAD), hyperlipidemia (HLD), hypertension (HTN), pulmonary embolus/deep vein thrombosis (PE/DVT), preexisting arrhythmia, pulmonary hypertension (pHTN), obesity, smoking, preexisting valvular disease, preexisting systolic HF, preexisting diastolic HF, beta-blocker use, ACE-inhibitor/ARB use, statin use, hydralazine/nitrate use, LGE and CMR circumferential and longitudinal strain. The six outcomes include MI, systolic HF, valvular disease, arrhythmia, and diastolic HF. With univariate regression analysis, each predictor is examined. Predictors were selected based on a *p*-value of less than 0.05. The selected predictors then were used to build the multivariate competing risk regression model. In survival analyses, an outcome of interest may prevent the observation of or affect the chance of another outcome of interest from occurring. Such an outcome is known as a competing risk and must be properly addressed to avoid incorrect conclusions. To avoid the bias in the Kaplan Meier method when there are multiple events or failures, a competing risk regression model was used [42].

### Ethical approval

Our study was approved by the Yale IRB committee. The IRB waived the requirement for informed consent as this was a retrospective review and no identifiers are used in reporting results.

## Results

Of the 116 female breast cancer patients who had received CMR for evaluation of either cardiomyopathy or cardiac symptoms included in this study, the median age was 66 years old. Majority of the patients were Caucasian (83%). In our sample, 62 were in the AT group and 54 were in the NAT group.

Patient demographic and clinical characteristics at the time of CMR are listed in **Table 1**. The AT group had a mean age of 62 years old, and the NAT group was older with a mean age of 68 years old (*p* = 0.001). The incidence of diastolic dysfunction by echo was higher in the NAT group compared to AT group (37 vs 16%) (*p* = 0.019).

**Table 1:** Patient characteristics according to Her2/ATC treatment status.

	AT	NAT	Total	P Value
	N = 62 (%)	N = 54(%)	N = 116 (%)	
**Age Mean (stdev)**	61.6±11.3	68.2±10.4	64.6±11.3	0.001*
**Group**				
<35	0(0.0)	0(0.0)	0(0.0)	<0.001*
36–49	7(11.3)	3(5.5)	10(8.6)	
50–64	27(43.5)	15(27.8)	42(36.2)	
65+	28(45.2)	36(66.7)	64(55.2)	
BMI >30; Obesity				0.680
<30	39(62.9)	31(57.4)	70(60.3)	
>30	23(37.1)	23(42.6)	46(39.7)	
Ethnicity				1.000
White	51(82.3)	46(85.2)	97(83.6)	
Black	8(12.9)	6(11.1)	14(12.1)	
Hispanic	2(3.2)	2(3.7)	4(3.4)	
Asian	1(1.6)	0(0.0)	1(0.9)	
Other	0(0.0)	0(0.0)	0(0.0)	
Comorbidities				
HTN	27(43.5)	33(61.1)	60(51.3)	0.089
DM2	14(22.6)	10(18.5)	24(20.7)	0.752
Stroke/PE/DVT	9(14.5)	9(16.7)	18(15.5)	1.000
CAD	11(17.7)	17 (31.5)	28(24.1)	0.132
PAD	2(3.2)	3(5.6)	5(4.3)	0.888
Arrhythmia: a fib, a flutter	8(12.9)	14(25.9)	22(19.0)	0.122
Bradycardia	1(1.6)	6(11.1)	7(6.0)	0.080
Heart block/BBB	3(3.2)	5(9.3)	8(6.9)	0.572
Ventricular Arrhythmia	9(14.5)	4(7.4)	13(11.2)	0.359
Combination arrhythmias	2(3.2)	6(11.1)	8(6.9)	0.192
Family History of heart disease	20(32.3)	24(44.4)	44(37.9)	0.247
HF: Systolic	8(12.9)	4(7.4)	12(10.3)	0.507
**HF: Diastolic**	**10(16.1)**	**20(37.0)**	**30(25.9)**	0.019*
HF: both	10(16.1)	8(14.8)	18(15.5)	1.000
Valvular Disease	10(16.1)	15(27.8)	25(21.6)	0.195
pHTN	4(6.5)	4(7.4)	8(6.9)	0.863
HLD	26(41.9)	27(50.0)	53(45.7)	0.493
CKD/ESRD	2(3.2)	4(7.4)	6(5.2)	0.554
**Smoking**				0.210
Never Smoker	40(64.5)	25(46.3)	65(56.0)	
Current Smoker	13(22.4)	17(31.5)	30(25.9)	
Former Smoker	9(13.8)	12(22.2)	21(18.1)	

Patients’ cardiac and cancer medications are included in **Table 2**. Cancer stage, estrogen, progesterone receptor, Her2 positivity are featured in **S2 Table in S1 File**. A greater number of AT patients had CMR performed for an indication of cardiomyopathy as compared with the NAT group (53.2% vs 16.7%, respectively, p = 0.002) S3 Table in S1 File. Among patients in the AT group, 43.5% had been treated with trastuzumab and/or pertuzumab, 83.9% had been treated with anthracyclines, and 31% had received both therapies. The majority of all patients received radiation therapy, with no difference between the groups (82.3% of AT vs 74.1% of NAT (*p* = 0.399). The majority of patients also received anti estrogens (52% of AT and 57% of NAT, p = 0.663). Immune checkpoint inhibitors and tyrosine kinase inhibitors were not commonly used treatments in this patient cohort.

**Table 2:** Patient cardiac and cancer medications according to Her2/ATC treatment status.

	AT	NAT	Total	P value
	N = 62 (%)	N = 54 (%)	N = 116 (%)	
*Cardiac Medications*				
Beta blocker	32(51.6)	36(66.7)	68(58.6)	0.146
ACEi/ARB	27(43.5)	20(37.0)	47(40.5)	0.603
Statins	27(43.5)	26(48.1)	53(45.7)	0.752
Nitrates	3(4.8)	2(3.7)	5(4.3)	0.888
*Cancer therapy*				
**Herceptin/Perjeta**	**27(43.5)**	**0(0.0)**	**27(23.3)**	< .0001*
ICI	1(1.6)	0(0.0)	1(0.9)	1.000
**Anthracycline**	**52(83.9)**	**0(0.0)**	**52(44.8)**	< .0001*
**TKI**	**6(9.7)**	**1(1.9)**	**7(6.0)**	**0.169**
Radiation Therapy	51(82.3)	40(74.1)	91(78.4)	0.399
*Other Cancer Therapies*				
PARP inhibitors	0(0.0)	0(0.0)	0(0.0)	1.000
**Anti-Metabolites**	**9(14.5)**	**1(1.9)**	**10(8.6)**	0.036*
SERMs/Anti-estrogens	32(51.6)	31(57.4)	63(54.3)	0.663
**Platinum Agents**	**5(8.1)**	**0(0.0)**	**5(4.3)**	0.094
Topoisomerase Inhibitors	0(0.0)	0(0.0)	0(0.0)	1.000
Proteasome Inhibitors	0(0.0)	3(5.6)	3(2.6)	0.196
**Alkylating agents**	**30(48.4)**	**0(0.0)**	**30(25.9)**	< .0001*
**Taxanes**	**45(72.6)**	**0(0.0)**	**45(38.8)**	< .0001*

### CMR function and volumes

CMR imaging was performed during cancer treatment in 30 patients (48%) in the AT group and in 20 patients (37%) in the NAT group, with no significant difference between the two groups (p = 0.218). The remainder of the studies were completed after chemotherapy. There was no significant difference in LVEF (AT 57% +/- 10.1 vs NAT 60% +/- 11.2, p = 0.077) nor LV mass index (AT 56 +/- 18 vs NAT 61 +/- 18, p = 0.207) between the two groups. The AT group had higher left ventricular end systolic volume indexed (LVESVI) compared to the NAT group (42 +/- 24 vs 29 +/- 18, *p* = 0.004). Right ventricular ejection fraction (RVEF) was lower in the AT group compared to the NAT group (54% +/- 9 vs 58% +/- 8, p = 0.04). Furthermore, reduced RVEF was found significantly more in the AT cohort compared to the NAT cohort (37.8% vs 13.5%, p = 0.014). The right ventricular end diastolic volume indexed (RVEDVI) was also higher in the AT group (85.2 = /-37) compared to the NAT group (69.8 +/-26.2), p = 0.004. The CMR LA volume was smaller in the AT vs the NAT group (45 mL +/- 20 vs 58 mL +/- 25, *p* = 0.003) **Table 3.**

**Table 3:** Cardiac magnetic resonance imaging parameters according to AT treatment status.

Parameters	AT	NAT	Total	P value
CMR LVEF (%)	57±10	60±11.2	59.3 ±11.0	0.077
CMR LVEDVI	70.5±18.7	66.8±21.0	68.8±19.6	0.313
**CMR LVESVI**	**41.6±24.3**	**29.7±17.8**	**36.2±22.4**	0.004*
CMR LVSV (mL)	73.0±18.0	72.9± 22.7	73.2±20.3	0.870
CMR LVSVI	38.2±9.7	39.8±11.5	38.9±10.6	0.419
CMR LV Mass Index	56.3±18.8	60.6±17.7	58.2±18.0	0.208
CMR CO (L/min)	5.3±1.3	4.8±1.3	5.0±1.3	0.106
CMR CI (L/min/m2)	2.8±0.7	2.6±0.6	2.7±0.6	0.052
**CMR RVEDVI**	**85.2±37.0**	**69.8± 26.2**	**77.9±30.0**	0.005*
CMR RVSV (mL)	71.7±1801	70.1±22.0	70.9± 19.9.7	0.6850
CMR RVSVI	38.3±7.9	37.7±10.2	38.0 ± 9.7	0.711
**CMR RVEF (%)**	**53.9±8.8**	**57.1±8.0**	**55.4± 9.0**	0.047*
CMR LVEDD (mm)	50.6±6.4	49.2±7.0	49.9 ± 6.8	0.240
**CMR LVESD (mm)**	**36.3±6.6**	**32.9±7.4**	**34.7± 7.4**	0.015*
CMR LA Size (cm2)	21.3±6.0	20.0±6.5	20.7± 6.3	0.216
CMR RA Size (cm2)	18.3±4.6	18.4±4.6	18.3± 4.6	0.856
**CMR LA Biplane Volume (cm3)**	**45.2±20.3**	**57.7±24.9**	**51.0± 23.3**	0.003*
**CMR Findings BSA**	**1.9±0.2**	**1.8±0.2**	**1.8± 0.2**	0.752*
CMR LAVAI Biplane	24.9±11.3	30.9±11.9	27.7±11.9	0.007
CMR GLS (%)	-12.6±816	-12.9±5.6	-12.9±5.6	0.789
CMS CS (%)	-17.2±7.3	-17.0±8.1	-17.2±7.6	0.899

### MRI GLS and GCS

Average GLS was reduced for both the AT and NAT cohorts, without a significant difference between the two groups (-12.6 vs -12.9, respectively, p = 0.789). Values for average GCS were borderline normal and not significantly different between the two groups (-17.2 vs-17.0, respectively, p = 0.899) (**Table 3**). In a sub-analysis of the AT group, GLS was similar in those who received anthracycline alone compared to those who received HER-2 inhibitors alone (GLS -13.0 vs -15.0, respectively, p = 0.095). However, GLS in those who received anthracycline alone was more abnormal compared to those who received both anthracycline and a HER2i agent (-13.1 vs -15.6 p = 0.02). However, GLS was not statistically significant between those who received both anthracycline and HER-2 inhibitors versus HER-2i alone (p = 0.803). Circumferential strain was similar amongst the groups in this sub-analysis of the AT cohort, with no statistical significance seen (**S1 Fig in S1 File**). Anthracycline use was associated with significantly more abnormal GLS (GLS >-18) (74.4%) compared to no anthracycline use (40%) (*p* = 0.036) (**Table 4**).

**Table 4:** Significant Pearson Correlations for MRI GLS, GCS, chi square analyses for AT vs NAT (a) and chi square analyses for LGE (b).

(a)									
Significant Correlations	Coefficient				P value				
*All Patients*									
MRI GLS (continuous) & MRI GCS (continuous)	0.49				0.000				
MRI GCS (continuous) & MRI LVEF (continuous)	-0.32				0.001				
MRI GCS (continuous) & MRI LVESVI (continuous)	0.22				0.021				
MRI GCS (continuous) & MRI LVEDVI (continuous)	0.18				0.06				
*AT Patients*									
MRI GLS (continuous) & MRI GCS (continuous)	0.493				0.001				
MRI GCS (continuous) & MRI LVEF (continuous)	-0.37				0.003				
	AT (N (%)		NAT N (%)		Total N (%)		P value		
*Abnormal GLS*	36 (67.9%)		28 (84.8%)		64 (74.4%)		0.080		
*Abnormal GCS*	21 (33.9%))		13 (24.1%))		34 (28.5%)		0.247		
*Abnormal LVEDVI*	16 (34.8%)		14 (37.8%)		301 (36.1%)		0.773		
*Abnormal LV mass index*	8 (17.4%)		6 (16.2%)		14 (16.9%)		0.887		
***LGE positivity***	**8 (13.3%)**		**14 (29.2%)**		22 (20.4%)		**0.042**		
***Reduced RVEF by CMR***	**17 (37.8%)**		**5 (13.5%)**		**22 (26.8%)**		**0.014**		
*AT group*	**Anthracycline(**N (%)		**No anthracycline** N (%)		Total N (%)		P value		
***Abnormal GLS***	**32 (74.4%%)**		**4 (40%)**		**36 (67.9%)**		** 0.036**		
*AT group*	**Her2i** (N (%)		**No Her2i** N (%)		Total N (%)		P value		
*Abnormal GLS*	14 (58.3%)		22 (75.9%)		36 (67.9%)		0.174		
(b)									
	***LGE positive***		***LGE negative***		***Total***		***P***		
*All patients*									
***Abnormal GLS***	***16 (94*.*1%)***		***48 (69*.*6%)***		***64 (74*.*4%)***		***0*.*038***		
***Abnormal GCS***	***11 (61*.*1%)***		***23 (29*.*1%)***		***34 (35*.*1%)***		***0*.*010***		
***Increased LV mass index***	***6 (37*.*5%)***		***2 (3%)***		***8 (9*.*6%)***		***0*.*0001***		
*Increased LVEDVi*	*5 (31*.*3%)*		*23 (34*.*3%)*		*28 (33*.*7%)*		*0*.*534*		
*Reduced RVEF*	*3 (18*.*8%)*		*19 (28*.*8%)*		*22 (26*.*8%)*		*0*.*416*		
***CAD***	***10 (45*.*5%)***		***14 (16*.*3%)***		***24 (22*.*2%)***		***0*.*003***		
***Valvular disease***	***8 (36*.*4%)***		***12 (14%)***		***20 (18*.*5%)***		***0*.*016***		
*PE/DVT*	*6 (27*.*3%)*		*10 (11*.*6%)*		*16 (14*.*8%)*		*0*.*065*		
*Heart failure*	*8 (36*.*4%)*		*17 (20%)*		*25 (23*.*4%)*		*0*.*106*		
*AT patients*									
*Increased LV mass index*	**2 (33.3%)**		**2 (5%)**		**4 (8.7%)**		**0.045**		
*Increased LVEDVi*	1 (2.5%)		1 (16.7%)		2 (4.3%)		0.088		
***CAD***	**4 50%)**		**5 (9.6%)**		**9(15%)**		**0.013**		
***Valvular disease***	**4 (50%)**		**5 (9.6%)**		**9 (15%)**		**0.013**		
*PE/DVT*	3 (37.5%)		5 (9.6%)		8 (13.3%)		0.065		

Among all patients, when compared with other CMR parameters, GLS significantly correlated with GCS (r = 0.493; *p* = 0.0001)). Additionally, GCS significantly correlated with LVEF (r = -0.32; *p* = 0.001) and LVESVI (r = 0.22; *p* = 0.021). In an AT group sub-analysis, GLS remained significantly correlated with GCS (r = 0.43; *p* = 0.001), and GCS was negatively correlated with LVEF (r = -0.37, *p* = 0.003) (**Table 4A**)

### Late gadolinium enhancement

In the overall cohort, 22 had LGE and there was significantly more LGE positivity in the NAT group compared to AT group (29% vs 13%, p = 0.042). In the AT cohort, 8 patients demonstrated LGE (13%), all in a nonischemic pattern, and one had no contrast given. In the NAT cohort, 14 (29%) had LGE, of which 11 (20%) were nonischemic, 3 (5%) were ischemic, and 2 (4%) had nondiagnostic LGE images. LGE positivity was significantly associated with abnormal GLS, GCS, increased LV mass index, and reduced RVEF. Other imaging parameters were not statistically significant and are listed in **Table 4 (bottom)**. Presence of LGE was also significantly correlated with clinical co-morbidities of CAD and valvular disease. In a subgroup analysis of the AT cohort, presence of LGE was associated with increased LV mass index, the presence of CAD, and valvular disease (**Table 4B**).

### Stress CMR

In this sub-analysis, 13 patients had stress CMR performed for cardiomyopathy evaluation while on chemotherapy (5 with anthracycline alone, 2 on herceptin alone and 4 received both). In this cohort, the average LVEF was 59.7% and average GLS was -15.12%. Three patients (23%) had perfusion defects and/or LGE consistent with ischemic heart disease (IHD), and 1 patient had a non-ischemic pattern of LGE, whom had prior anthracycline exposure. The average sub-endocardial/sub-epicardial myocardial perfusion index ratio (SS-MPIR) at stress was 0.98 for all patients, which was normal based on published references [43]. When all ischemic segments were excluded, it was 1.00, and when all patients with IHD were excluded, the SS-MPIR was 1.02. CMR LVEF correlated with SS-MPIR when all segments were analyzed (r = 0.899, *p* < 0.01), when ischemic segments were excluded (r = 0.643, *p* = 0.024), and when patients with IHD were excluded (r = 0.807, *p* = 0.009). No significant correlation was found between different breast cancer therapies and CMR SS-MPIR or GLS.

### Cardiovascular outcomes comparing AT vs NAT

**Fig 4:**
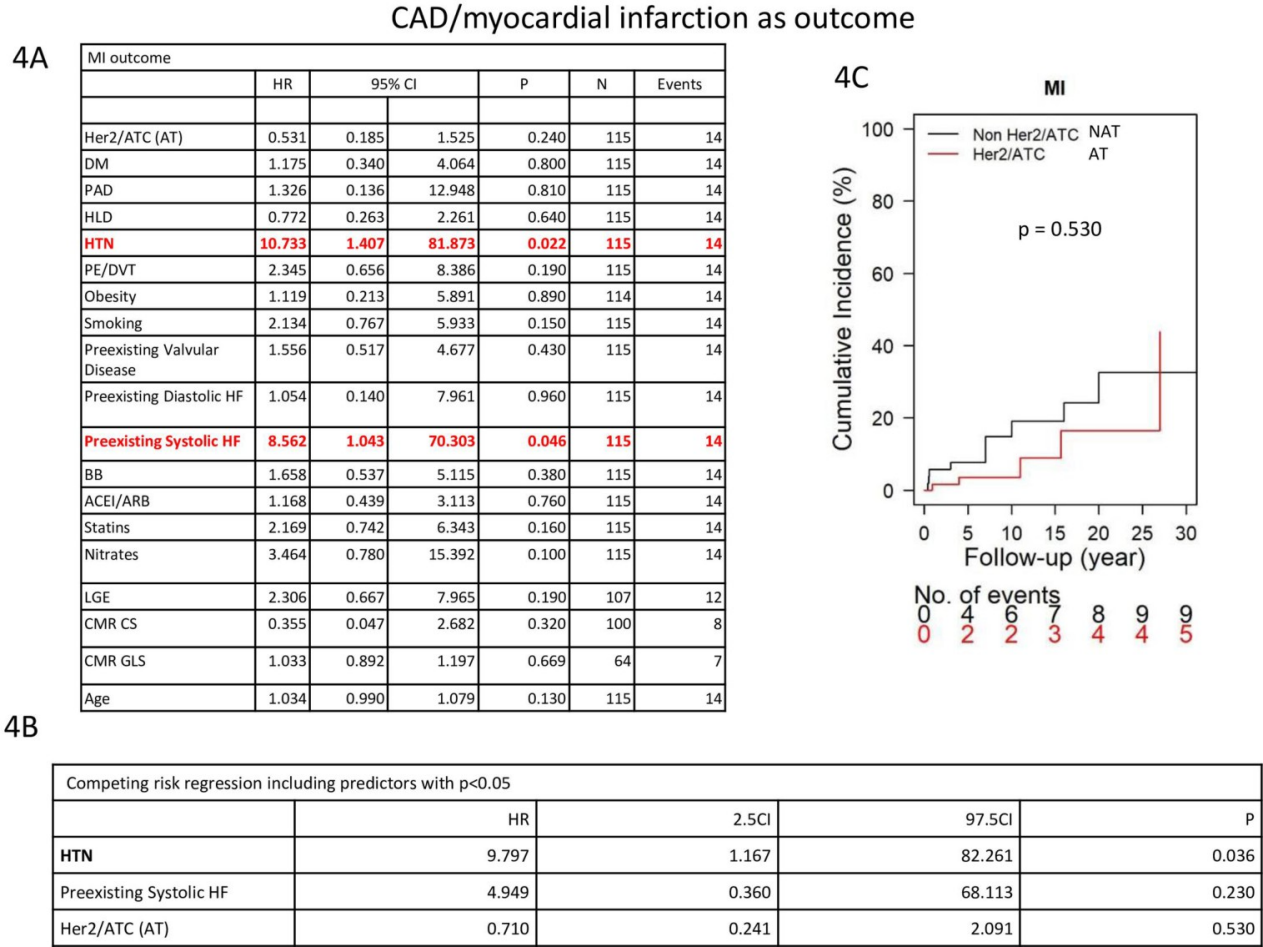
Competing risk regression model evaluating factors associated with CAD/Myocardial Infarction.

**Fig 5:**
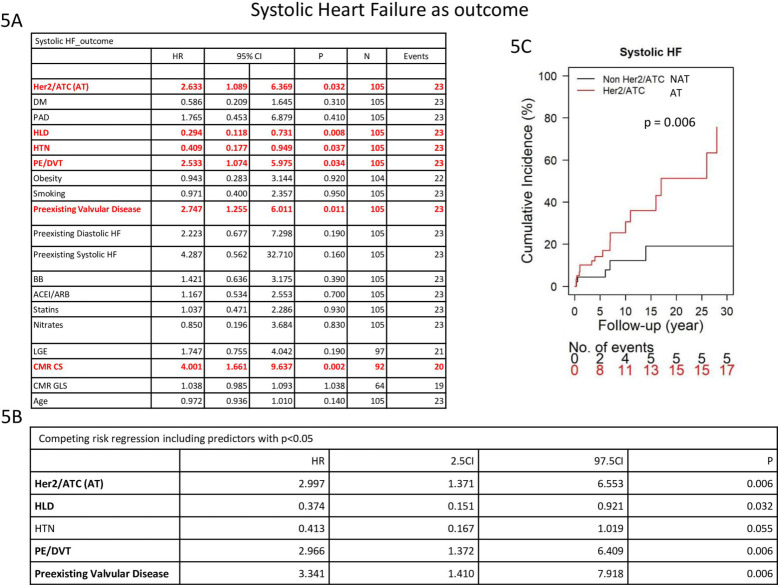
Competing risk regression model evaluating factors associated with systolic heart failure.

**Fig 6:**
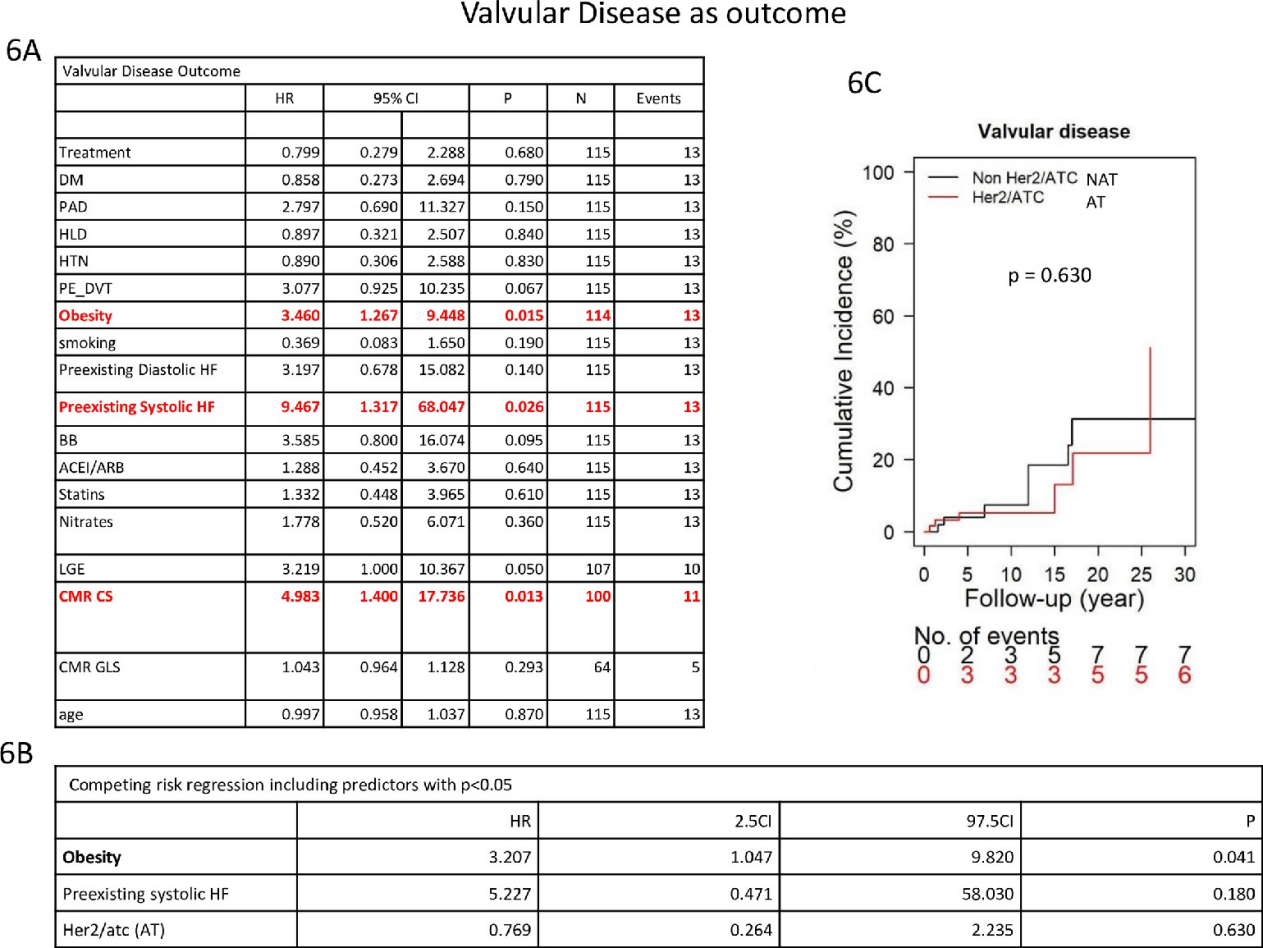
Competing risk regression model evaluating factors associated with valvular disease.

**Fig 7:**
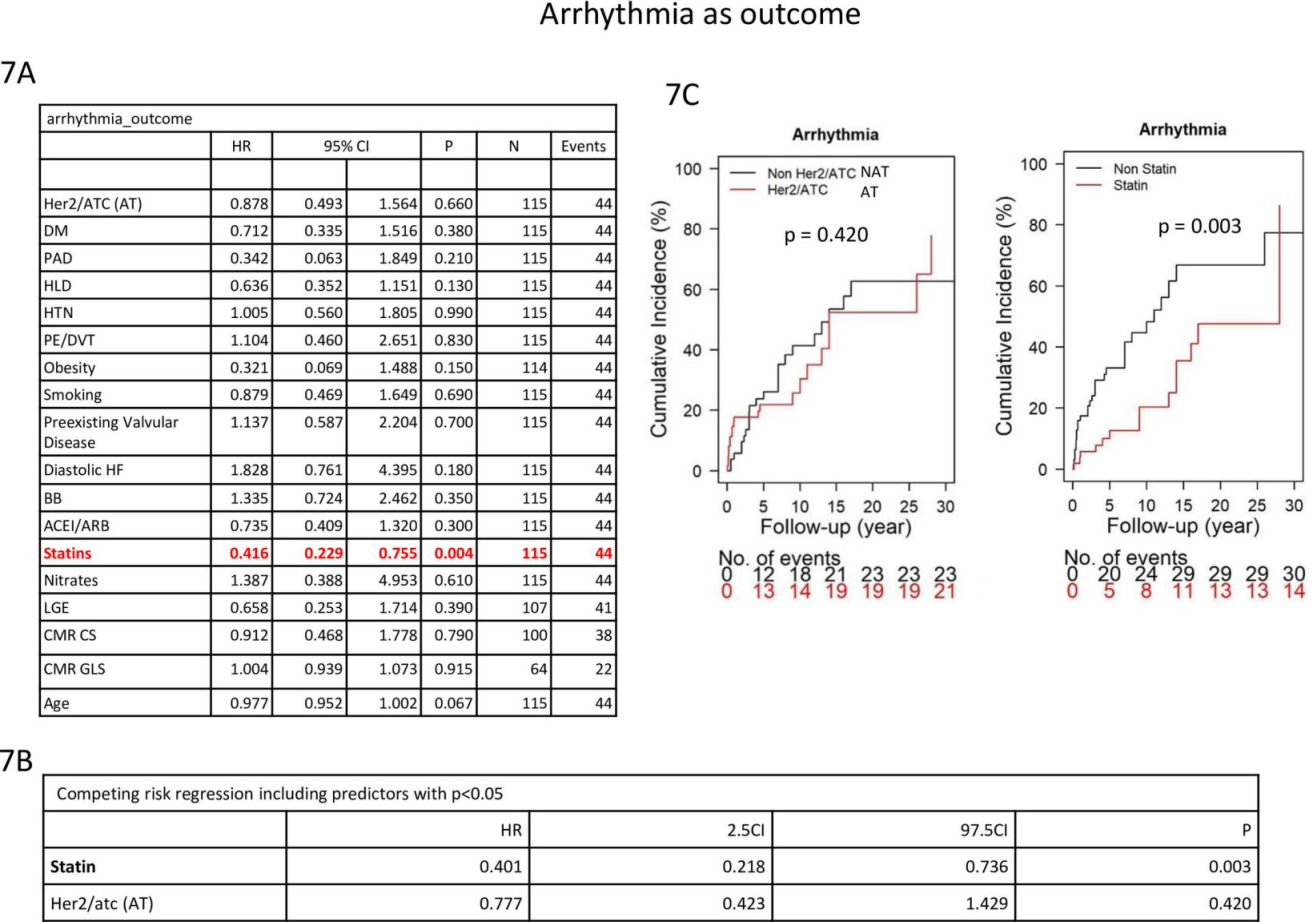
Competing risk regression model evaluating factors associated with arrhythmia.

Cardiovascular outcomes between the AT and NAT cohorts were evaluated. The AT cohort had an increase in systolic heart failure outcome compared with the NAT cohort (27.4% vs 10.9%, p = 0.025) (**Table 5**). There is a significant difference in regards to systolic heart failure, with the AT group having a higher incidence of systolic heart failure over time, *p* = 0.006, **Fig 5C**. No significant differences in outcomes were seen between the two groups for CAD/myocardial infarction (*p* = 0.530) (**Fig 4**), valvular disease (*p* = 0.630) (**Fig 6**), arrhythmia (*p* = 0.42) (**Fig 7**) or diastolic heart failure (p = 0.16) (**S2 Fig in S1 File**).

**Table 5:** Cardiovascular outcomes between AT and NAT groups.

Outcomes	AT N (%)	NAT N (%)	Total N (%)	P Value
**MACE (HF/CAD/MI/arrhythmia, death)**				
**HF: Systolic**	**17 (27.4)**	**6 (10.9)**	**23 (19.7)**	0.025*
HF: Diastolic	14 (22.6)	16 (29)	30 (25.6)	0.421
CAD/MI	7 (11.3)	9 (16.3)	15 (12.8)	0.425
Arrhythmia	21 (33.8)	23 (41.8)	44(37.6)	0.376
Valvular disease	6 (9.7)	7 (13.4)	13 (11.1)	0.527
Death	3 (4.8)	4 (7.7)	7 (6.0)	0.579

Amongst the entire cohort of 116 breast cancer patients, the relationship between cardiovascular risk factors and cardiovascular outcomes was evaluated. Hypertension and preexisting systolic heart failure (Fig 4A) were univariately associated with an increased risk of CAD/ myocardial infarction (HR: 10.73, CI: 1.41–81.873, *p* = 0.022 and HR:8.56, CI: 1.04–70.30, *p* = 0.046, respectively) (**Fig 4**). Competing risk regression showed that HTN was significantly associated with a 9.8-fold increase in developing CAD/myocardial infarction (**Fig 4B)**. Cumulative incidence over time is featured in **Fig 4C**.

In univariate analysis, use of anthracycline and/or Her2i (HR: 2.63, CI 1.089–6.369, *p* = 0.032), PE/DVT (HR:2.53, CI: 1.07–5.96, *p* = 0.034), pre-existing valvular disease (HR:2.75, CI: 1.26–6.01, *p* = 0.011) and abnormal CMR GCS (HR:4.00, CI: 1.66–9.64, *p* = 0.002) were significantly associated with increased risk of developing systolic heart failure. However, HLD (HR:0.29, CI: 0.12–0.73, *p* = 0.008) and HTN (HR:0.41, CI: 0.18–0.95, *p* = 0.037) were associated with reduced incidence of systolic heart failure **Fig 5A**. Competing risk regression found that use of anthracycline and/or Her2i, PE/DVT and pre-existing valvular disease were independently associated with the systolic heart failure outcome, whereas HLD was negatively associated **Fig 5B**.

In the univariate analysis for the valvular disease outcome, obesity (HR: 3.46, CI: 1.27–9.45, *p* = 0.015), preexisting systolic HF (HR:9.47, CI: 1.32–68.05, *p* = 0.026) and CMR GCS (HR:4.98, CI: 1.40–17.74, *p* = 0.013) were significantly associated with increased risk **Fig 6A**. However, in the competing risk model, only obesity remained significantly correlated (HR: 3.2, CI 1.047–9.82, *p* = 0.041) **Fig 6B**. Cumulative incidence of valvular disease over time is featured in **Fig 6C**.

Statin use was significantly associated with reduced risk in the univariate model (HR: 0.416, CI: 0.229–0.755, *p* = 0.004) **Fig 7A** and in the competing risk regression model for arrhythmia (HR: 0.401, CI 0.218–0.736, *p* = 0.003) **Fig 7B**. Cumulative incidence of arrhythmia over time is featured in **Fig 7C**.

None of the predictors were significant for the diastolic heart failure outcome (**S2 Fig in S1 File),** as shown in the univariate analysis S1A Fig in S1 File. Cumulative incidence of diastolic dysfunction is shown in S1B Fig in S1 File.

**Fig 8:**
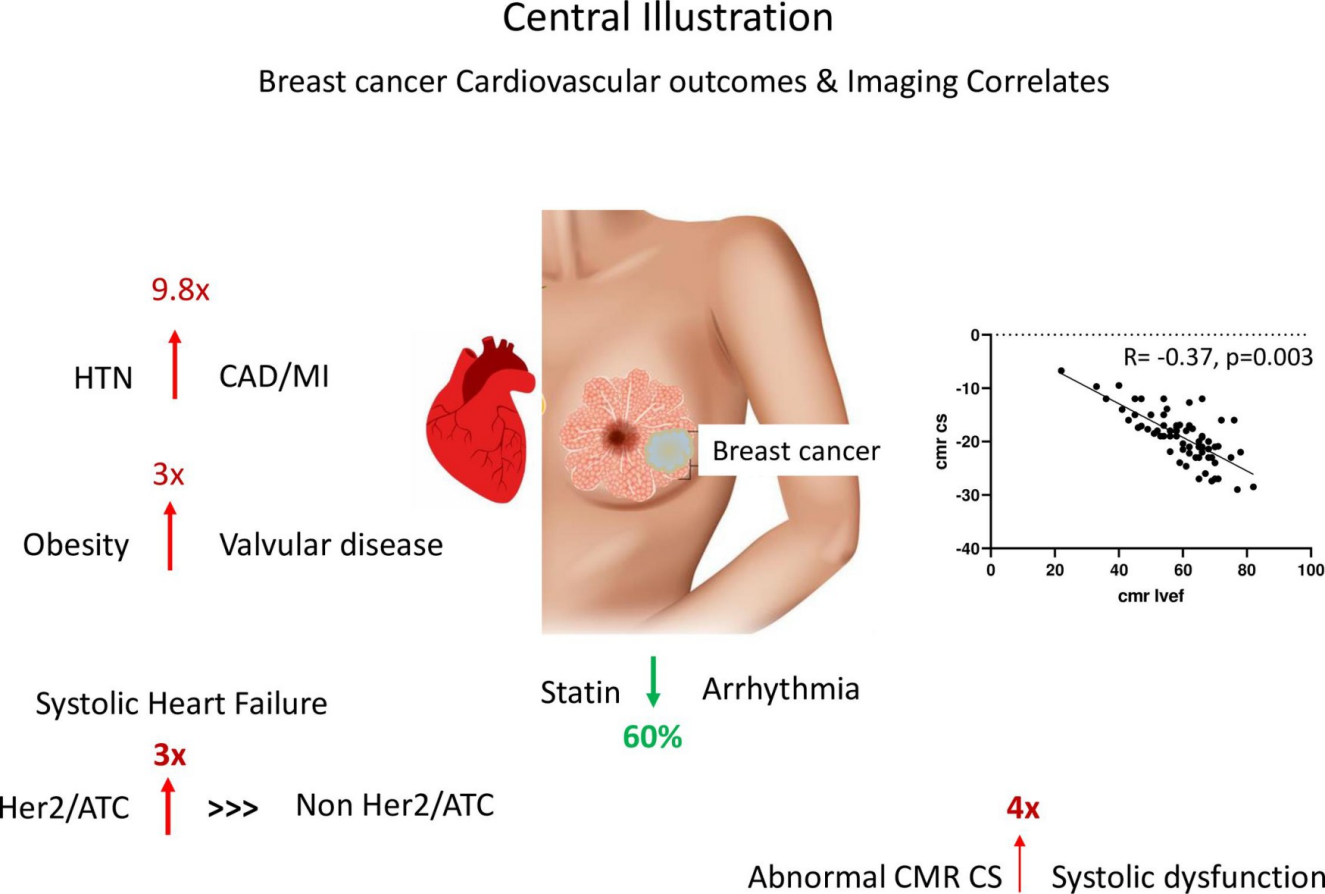
Central illustration summarizing key findings.

Of note, abnormal GLS and LGE were not independently associated with any of the above cardiovascular outcomes. A summary of our key findings is featured in **Fig 8.**

## Discussion

Leveraging comprehensive information on patient clinical and demographic characteristics, cardiovascular outcomes, and cardiac imaging parameters, we evaluated breast cancer patient outcomes comparing those on AT vs NAT. Our results provide information that may be used to help predict adverse cardiovascular outcomes in breast cancer patients who are treated with anthracyclines and trastuzumab. Furthermore, the information we obtained on the baseline characteristics can be useful in risk assessment of patients prior to initiating cardiotoxic chemotherapy and for monitoring of high-risk patients.

Our study has several implications for patients treated with breast cancer. First, our findings suggest the importance of a comprehensive evaluation prior to starting cancer therapy. Physicians and patients should consider family history, smoking, obesity and other cardiovascular comorbidities at baseline, as these may affect the risk of adverse cardiac outcomes during or after cancer therapy. It is important for oncologists to be aware of such cardiac risk factors and consider cardiology evaluation and monitoring when present. A recent study found that only 46.2% of breast cancer patients received the recommended cardiac monitoring while on cancer therapy [10].

In terms of cardiac function and volumes, the AT group had higher LV volumes, lower RVEF, and larger RVEDVi compared to the NAT group, but no significant difference in LVEF. These findings warrant further investigation to understand why AT may promote more adverse remodeling in the RV. Although cardioprotective medications, like beta blockers and ACE inhibitors, may affect recovery of cardiac function, there was no significant difference in the cardiac medication use between the two groups.

We found that strain was more abnormal in those who received anthracycline alone as compared to those who received both anthracycline and HER2i. Also, strain trended towards being more abnormal in those who received anthracycline alone versus her2i alone. Although there was no significant difference in age between these AT and Her2i groups, the AT group had more diastolic dysfunction at baseline. Another possible contributing factor to the difference between the groups is that patients who received both AT and HER2i may have been more closely monitored [44]. Further, there were more patients with LGE (both ischemic and nonischemic) in the NAT group, which could be due to the patients being older. For the most part, even though there weren’t any significant differences in cardiac co-morbidities between the AT and NAT cohorts, there was increased diastolic dysfunction in the NAT group. The small sample size and varying times from start of oncologic therapy to imaging limits greater generalizability, and a prospective study with serial evaluations would be needed to confirm whether these findings hold true. In the stress CMR sub-analysis, GLS was abnormal in the entire cohort, even though LVEF was normal at the time of imaging, which could represent subclinical LV dysfunction [45]. Additionally, stress CMR was helpful in distinguishing ischemic versus nonischemic causes of cardiomyopathy in patients actively undergoing cancer treatment. Although stress CMR has been shown to assess for flow reserve and microvascular dysfunction based on the flow gradient between the endocardial and epicardial layer as evidenced by a decreased global (SS-MPIR) [46], we did not find evidence of microvascular dysfunction in this cohort after adjusting for ischemic heart disease. One possible reason for this is that stress CMR imaging was typically performed after identification of cardiomyopathy and initiation of cardioprotective meds and LV function had normalized by the time of the exam. Additionally, the small sample size with stress CMR limits evaluation for statistical significance of these findings.

In our univariate and competing risk models, we found that abnormal CMR GCS was significantly associated with the outcomes of systolic heart failure and valvular disease. Thus, further research using CMR strain to predict those who may develop heart failure or valvular disease should be studied in a larger prospective cohort.

AT has been associated with increased risk of systolic heart failure in prior studies, by several fold [11–13], which our study confirms; and, a competing risk regression analysis shows that other risk factors can independently increase risk, including preexisting valvular disease and PE/DVT. Venous thromboembolism could be a surrogate for a more severe cancer burden and its incidence has been shown to be increased by heart failure itself [47]. It is unclear why a history of HLD was negatively associated with systolic heart failure as an outcome. One possibility is that patients were on cardiac medications that could have lent a cardioprotective effect.

Statins have been previously shown to have anti-arrhythmic effects in those with heart disease or post operatively for cardiac procedures in preventing atrial fibrillation and atrial flutter [48, 49]. Our study found that there is an association between statin use and decreased arrhythmia (including atrial fibrillation and atrial flutter) in this breast cancer cohort, and a prospective study is warranted to confirm this finding. More recently, although in lymphoma patients, the randomized controlled trial STOP CA showed that statins used for primary prevention reduced cardiomyopathy compared with placebo [50]. Thus, for breast cancer patients who have high cholesterol or elevated ASCVD score, there should be a low threshold for starting a statin.

Our findings are generally consistent with the previous literature on cardiovascular disease in breast cancer patients and we also elucidate some novel findings that may impact management of breast cancer patients, such as evaluating CMR GCS, consistent with the 2022 ESC guideline recommendation of monitoring for cardiotoxicity with GCS by CMR [15]. Although echo is first line based on the guidelines due to ease of access, CMR can be complementary when echo quality is suboptimal, for evaluation or verification of an accurate ejection fraction, and when tissue characterization for assessment of inflammation or fibrosis is warranted [51]. Further, AT may be associated with longer term adverse remodeling as evidenced by larger left and right ventricle sizes. The significance of this is unclear and further longitudinal follow up is needed. Of note, the cardiovascular risk factor of hypertension significantly increased risk of future CAD/MI, and thus aggressive risk factor management in this population is of critical importance.

### Study limitations

Limitations of this study include being a single center study and having a relatively small sample size. Thus, the ability to assess differences in outcomes of those on certain cancer therapies was limited, and future studies with larger cohorts may help elucidate this further. Additionally, most of our patients were Caucasian, which reduces generalizability to other ethnicities. The study was retrospective in nature, so there may exist factors not accounted for in the analysis, and there may be missing information with respect to cardiac risk factors. Given that we went back to the 1990s for patients’ first exposure to chemotherapy, this can introduce a lead time bias and confounders from differences in treatment approaches, including dosages that have evolved over time. Thus, prospective studies would be valuable to further explore the findings of this study. Furthermore, there may be selection bias for those who received anthracycline and/or her2 inhibitor compared to those who did not, such as age, comorbidities, and stage of breast cancer. Additionally, CMR imaging did not include T1 mapping for assessment of diffuse fibrosis or extracellular volume fraction in this cohort of patients, limiting the CMR assessment of tissue characterization that can be demonstrated with this more contemporary quantitative technique.

## Conclusions

CMR and CMR strain evaluation are important imaging tools to assess breast cancer patients for chemotherapy induced cardiotoxicity. Although patients may have preserved LVEF, many have abnormal strain, suggesting subclinical cardiotoxicity. Statin use may be associated with a reduction in future arrhythmias in this patient cohort.

### Clinical perspectives

#### Competencies in medical knowledge

In patients treated with cancer therapies, clinicians should be cognizant of cardiac diseases as a major side effect. Clinicians should be cautious when treating cancer patients who have cardiovascular risk factors, such as hypertension, and obesity,

#### Competency in patient care

A breast cancer patient, prior to starting and while on chemotherapy, should receive a comprehensive cardiac evaluation, including cardiac imaging with echocardiography, as well as cardiac magnetic resonance imaging when indicated. Breast cancer patients should be aware of the cardiovascular risks associated with cancer therapy and the need for thorough cardiac risk assessment and management. Cardiac monitoring should be an integral component of quality cancer care.

## Supporting information

S1 File (PPTX)

S1 Data (XLSX)

## Abbreviations

ATC: anthracycline
AT: Anthracycline/trastuzumab
ASCVD: Atherosclerotic Cardiovascular Disease
BB: Beta Blocker
BSA: Body Surface Area
CAD: Coronary Artery Disease
CKD: Chronic Kidney Disease
CMR: Cardiac Magnetic Resonance Imaging
CO: Cardiac Output
DVT: Deep Vein Thrombosis
ECV: Extracellular Volume
ESRD: End Stage Renal Disease
GCS: Global Circumferential Strain
GLS: Global Longitudinal Strain
GRS: Global Radial Strain
ATC/ Her2i: Anthracycline/ Trastuzumab +/- Pertuzumab
Her2i: Her2 inhibitor
HLD: Hyperlipidemia
HTN: Hypertension
IHD: Ischemic Heart Disease
LAVAI: Left atrial volume area index
LGE: Late Gadolinium Enhancement
LVEDD: Left ventricular end diastolic diameter
LVEDVI: Left ventricular end diastolic volume index
LVEDV: Left ventricular end diastolic Volume
LVEF: Left Ventricle Ejection Fraction
LVESD: Left Ventricle
LVSV: Left Ventricular Stroke Volume
LVESVI: Left Ventricular Stroke Volume Index
MI: Myocardial Infarction
MRI: Magnetic Resonance Imaging
NAT: Non Anthracycline/trastuzumab
NSTEMI: Non-ST segment elevated myocardial infarction
PAD: Peripheral Artery Disease
PE: Pulmonary Embolism
pHTN: Pulmonary Hypertension
RVEDV: Right Ventricular End Diastolic Volume
RVEF: Right Ventricular Ejection Fraction
RVSV: Right Ventricular Stroke Volume
RVSVI: Right Ventricular Stroke Volume Index
SSFP: Steady state free precession
ss-GRE: Stress-only perfusion
ss-MPIR: Steady state magnetization preparation inversion recovery
TTE: Transthoracic Echocardiogram
