# Supplementary figures and images for: Quantitative cardiovascular magnetic resonance findings and clinical risk factors predict cardiovascular outcomes in breast cancer patients
Source: PLoS One. 2023 May 30;18(5):e0286364. doi: 10.1371/journal.pone.0286364 (PMC10228774; doi:10.1371/journal.pone.0286364)

## Slide 1
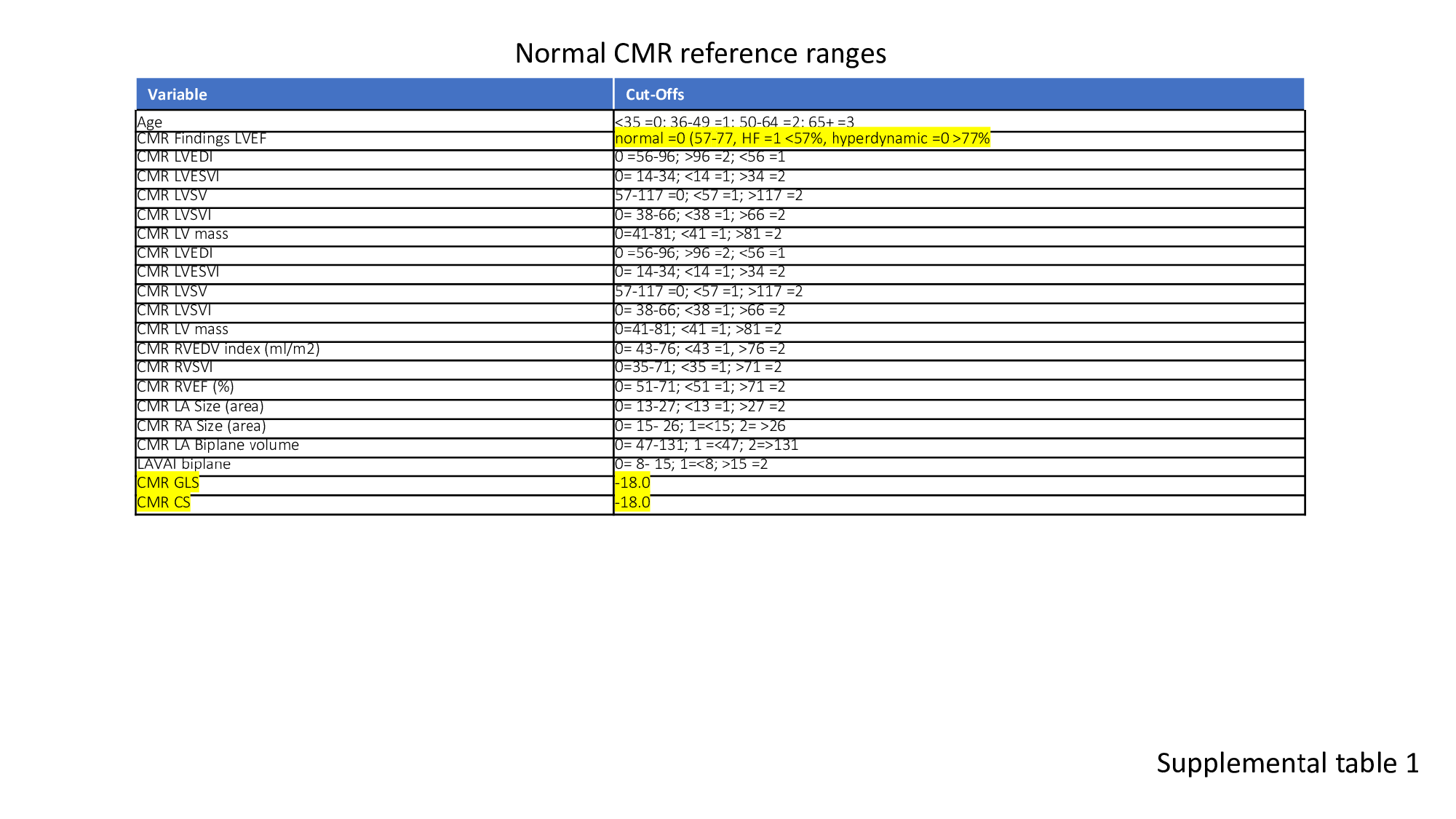

## Slide 2
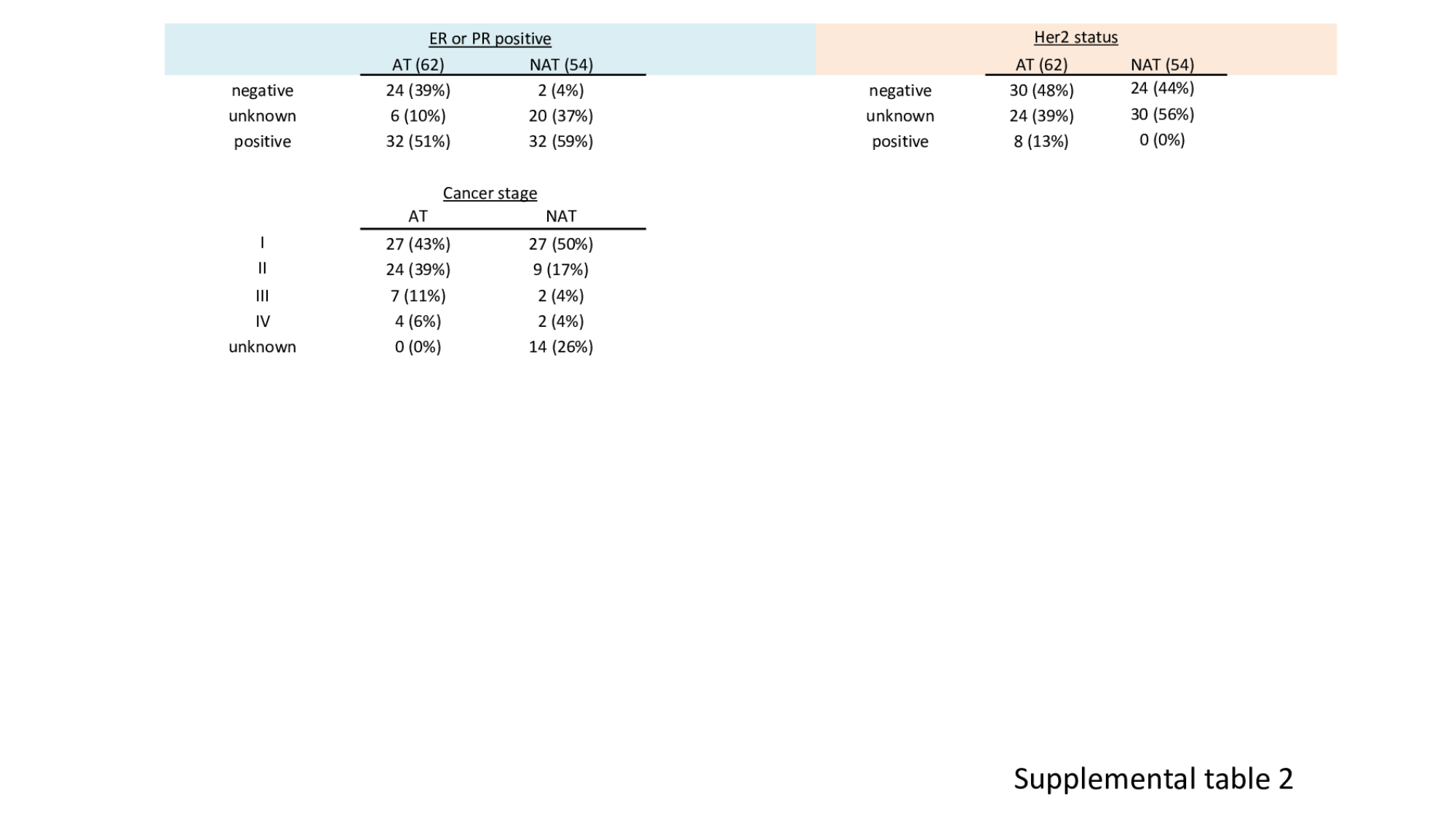

## Slide 3
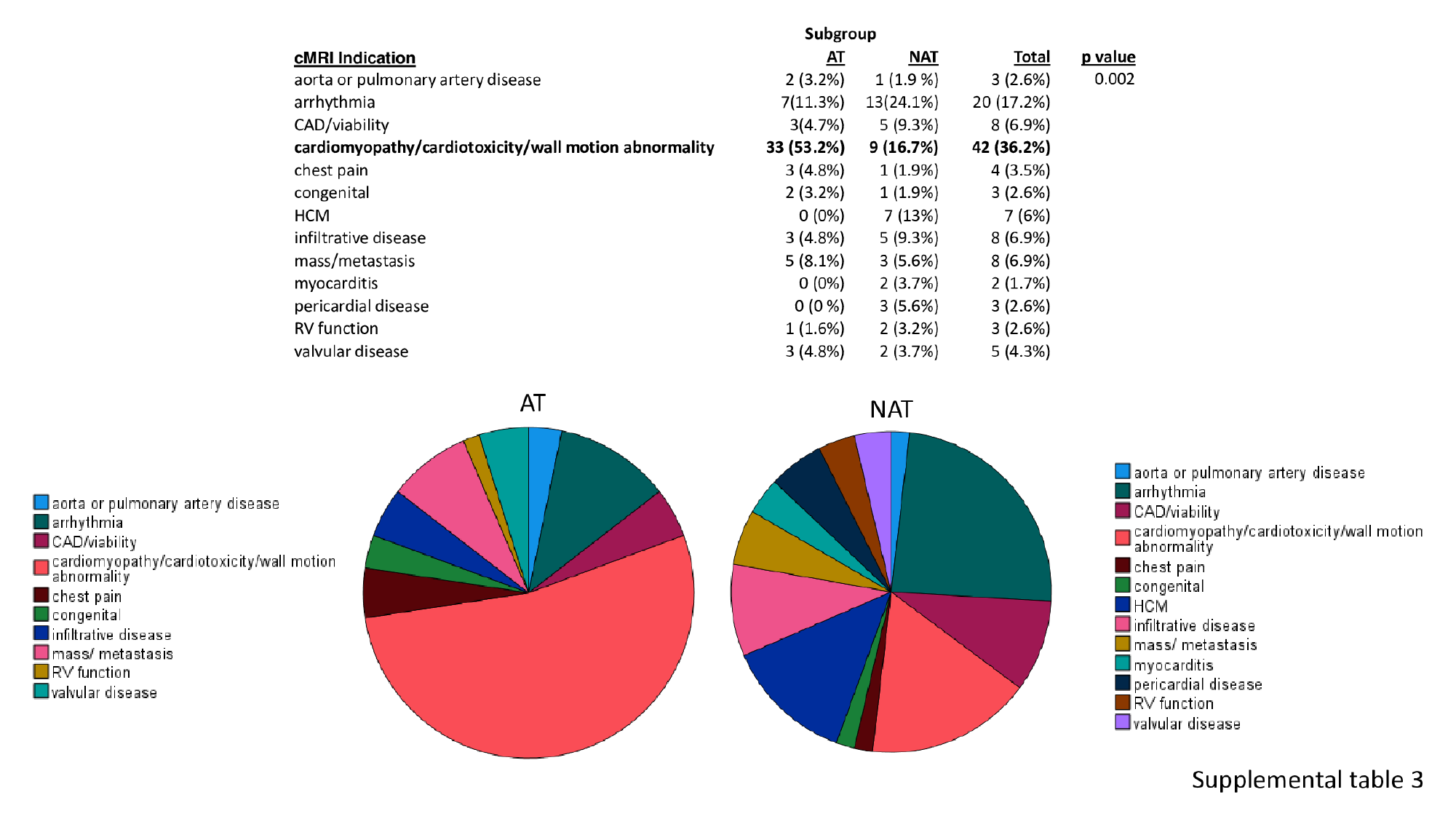

## Slide 4
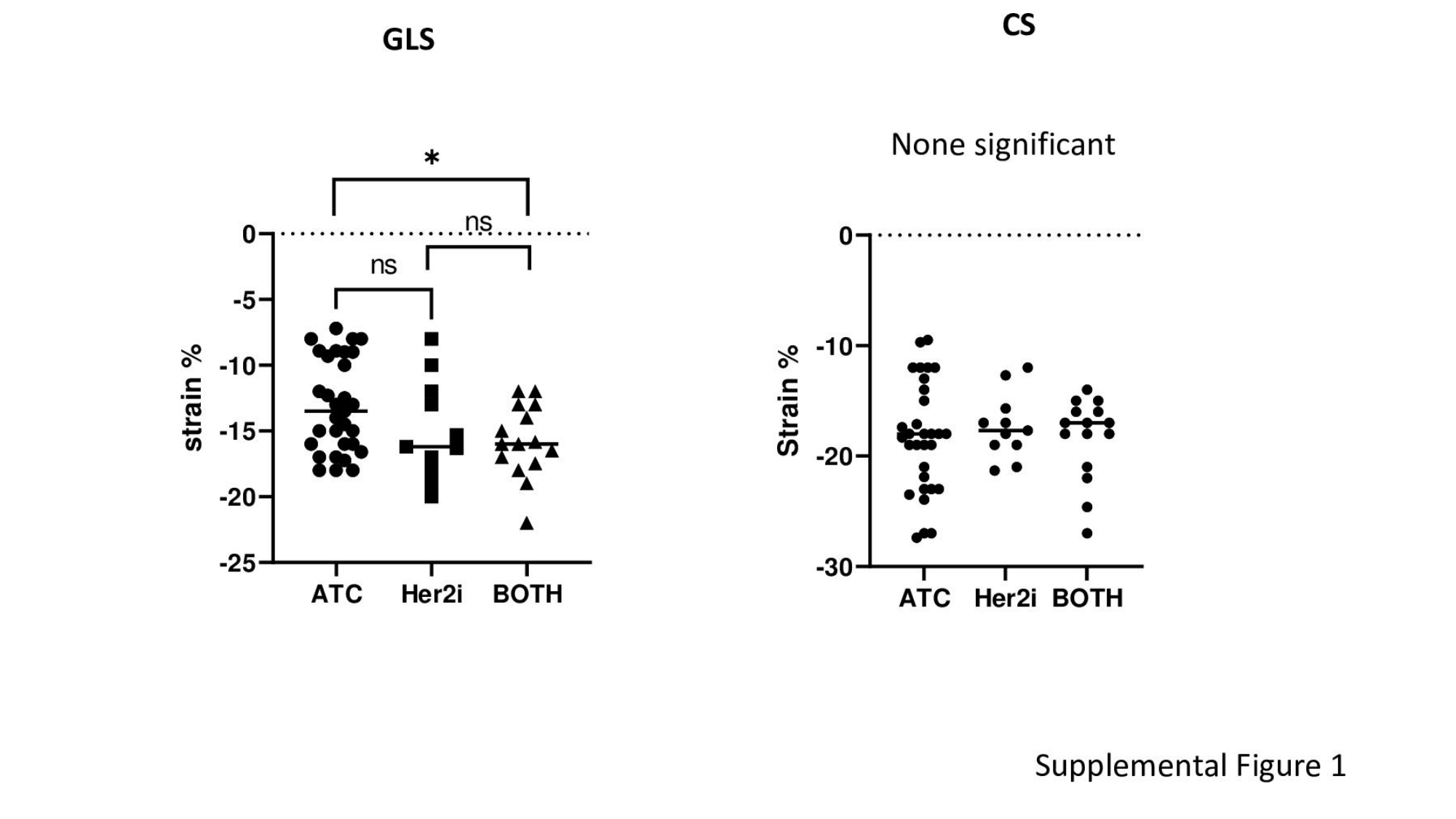

## Slide 5
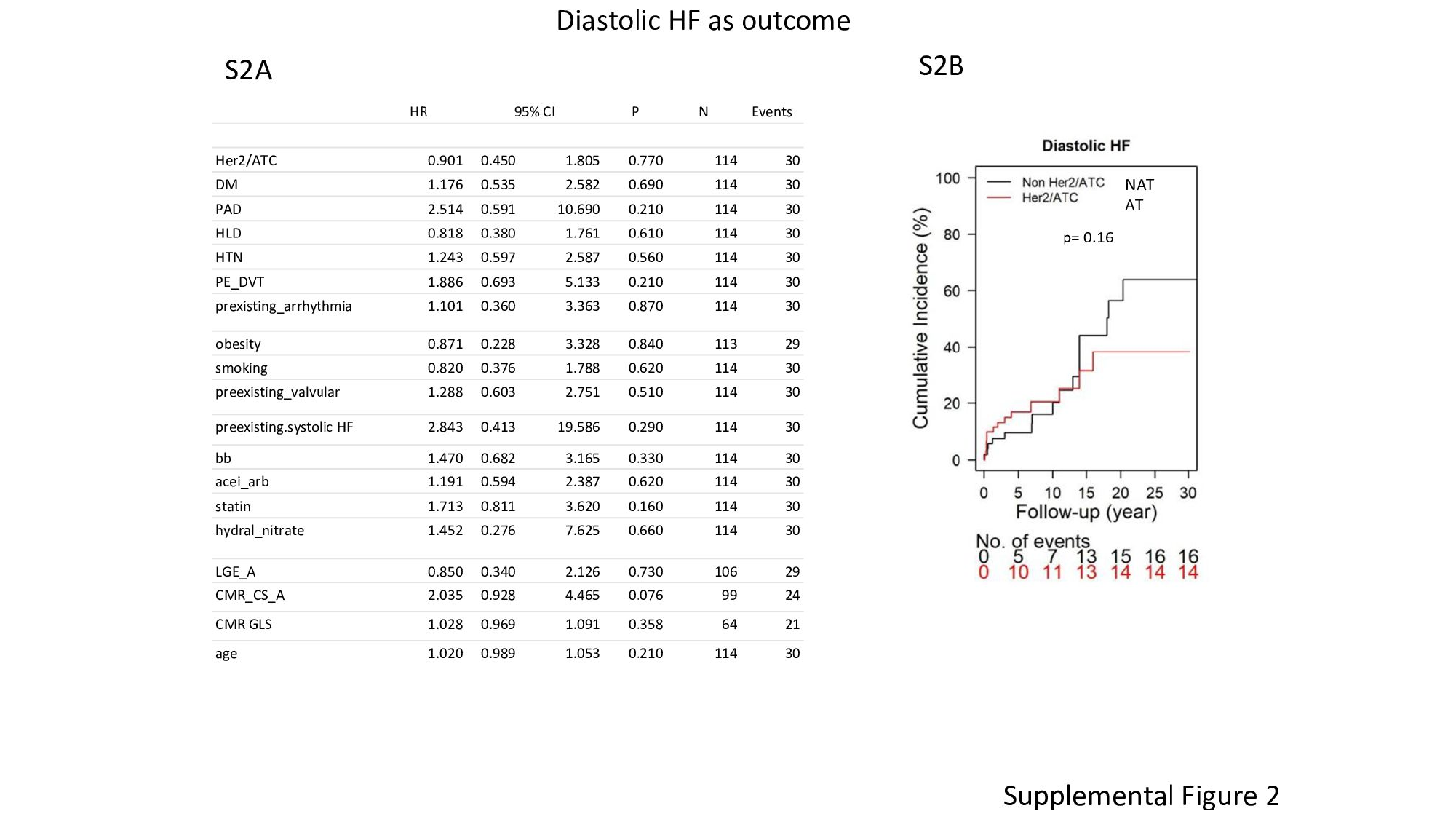

Supplement: S1 File — (PPTX) [file pone.0286364.s001.pptx]
